# Supplementary material for: Dissecting the Functional Interplay Between Heme Oxygenase LjHO1 and Leghemoglobins in Lotus japonicus Nodules
Source: Biology (Basel). 2025 Oct 13;14(10):1401. doi: 10.3390/biology14101401 (PMC12561041; doi:10.3390/biology14101401)
Supplement: Supplementary file 1 [file biology-14-01401-s001.zip › biology-3874769-supplementary.pdf]

Supplemental Figure S1

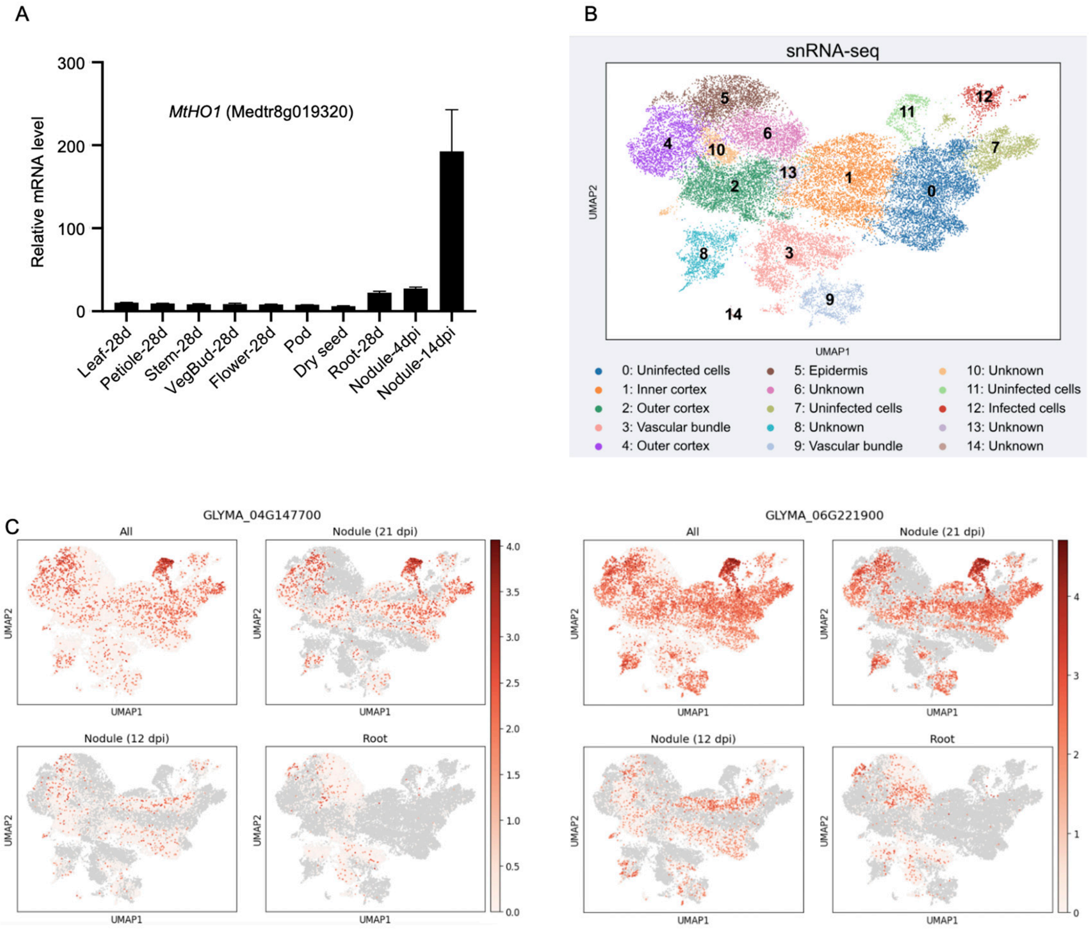

Figure S1. Expression profiles of heme oxygenase genes in *Medicago truncatula* and *Glycine max* derived from public datasets. (A) Relative mRNA level of *MtHO1* in different tissues of *M. truncatul*a. Data were retrieved from the database (https://lipm-browsers.toulouse.inra.fr/pub/expressionAtlas/app/mtgeav3/00.reference_dataset/Mtr.5569.1.S1_at). (B-C) UMAP (Uniform Manifold Approximation and Projection) visualization of 15 identified cell clusters (B) [1] and the expression patterns of *GmHO1* (GLYMA_04G147700) and *GmHO3* (GLYMA_06G221900) in the single-nucleus transcriptomes of soybean nodules and roots.

References

1. Liu, Z.; Kong, X.; Long, Y.; Liu, S.; Zhang, H.; Jia, J.; Cui, W.; Zhang, Z.; Song, X.; Qiu, L.; Zhai, J.; Yan, Z., Integrated single-nucleus and spatial transcriptomics captures transitional states in soybean nodule maturation. *Nature plants* **2023,** 9, (4), 515-524.

Table S1 Primers used in this study

Primer name	Sequence (5' to 3')	Purpose
LjHO1pro-F	GATCTACAGCGCTGACTTTACAGGATGCCTCACATG	Construction of *pLjHO1:GUS*
LjHO1pro-R	ACTGACCACCCGGGGGTTGATAGGGTTGAGTGCAC	
LjHO1-gRNA1-F	GTTCGATTGAGCGGAACTGACGATG	Construction of CRISPR plasmid
LjHO1-gRNA1-R	AAACCATCGTCAGTTCCGCTCAATC	
LjHO1-gRNA2-F	GTTCGAAACGACCTTGCTGTCAACG	
LjHO1-gRNA2-R	AAACCGTTGACAGCAAGGTCGTTTC	
LjHO1-CDS-*Bam*HI-F	AGT*<u>GGATCC</u>*ATGGCGTCAGCCACAGTTG	Subcellular localization in *L. japonicus* nodules
LjHO1-CDS-*Sma*I-R	CAC*<u>CCCGGG</u>*TGATAGAATTAGACGCAGAATCTC	
LjHO1pro-*Hind*III-F	GACGGCCAGTGCC*<u>AAGCTT</u>*CTTTACAGGATGCCTCAC	
LjHO1Pro-*Bam*HI-R	TGGCTGACGCCAT*<u>GGATCC</u>*GGTTGATAGGGTTGAGTG	
LjHO1TP-*Bam*HI-R	GCGGTACCGACAT*<u>GGATCC</u>*CGTCGCCGACACAATAAC	

Note: Restriction enzyme recognition sites are italic and underlined.
